# Motivation: Not as easy as it seems

**DOI:** 10.3205/zma001822

**Published:** 2026-02-17

**Authors:** Marjo Wijnen-Meijer

**Affiliations:** 1 TUD Dresden University of Technology, Medical Faculty and University Hospital Carl Gustav Carus, Institute of Medical Education, Dresden, Germany

## Editorial

The medical school curriculum is characterised by high demands and a long duration. Motivation plays a particularly important role in academic success [1], [2], [3], [4]. Current research examines various aspects of motivation and its influence on academic performance. Motivation can be understood as both a dependent and an independent variable. Motivation is studied as a dependent variable when it comes to identifying the factors that influence motivation. These include, for example, the learning environment, teaching methods and exam stress. A supportive learning atmosphere and teaching strategies that promote autonomy can increase motivation [5].

As an independent variable, motivation in turn influences other outcomes, particularly academic performance, learning strategy and long-term professional commitment. High intrinsic motivation promotes sustainable learning, while extrinsic motivation tends to favour short-term, results-oriented learning [6].

The type of motivation therefore has a direct influence on how students deal with challenges and how well they can develop skills in the long term. During the course of medical school, there is a significant decline in motivation, particularly in the areas of “willingness to make sacrifices” and “perseverance”. However, it should be noted that this decline does not correlate with exam results. This suggests a complex relationship between motivation and academic performance [1].

Students who participated in hybrid learning formats, i.e. a combination of face-to-face and online teaching, developed strong confidence in their own abilities and, as a result, a high level of self-efficacy. Factors such as gender, professional activity alongside their studies and involvement in extracurricular activities were found to be associated with higher self-efficacy. This suggests that not only learning content, but also, for example, the design of the learning environment has an important influence on student motivation [7].

A Brazilian long-term study conducted over a period of 30 months showed that medical students' motivation changed significantly during the course of medical school. While demotivation and external (extrinsic) motivation increased, internal (intrinsic) motivation decreased continuously. This decline in internal motivation and increase in demotivation was particularly noticeable in the first years of study [4].

Teaching approaches based on self-determination theory specifically promote intrinsic motivation by strengthening autonomy, competence and social integration. This can increase motivation and commitment to learning, reduce stress and improve mental well-being in the long term [3]. The motivation of medical students can be described as a dynamic process that is influenced by various factors. Although high autonomous motivation correlates with better academic performance, fluctuations occur during the course of study that are not always directly related to academic results. These findings underscore the importance of individual support and targeted fostering of motivation.

Teachers have a wide range of opportunities to encourage student motivation in all phases of training, both pre-clinical and clinical.

In the theoretical and pre-clinical phase:

- Promoting autonomy: Students benefit from having choices in learning paths, topics, or exam formats. Autonomy promotes the development of intrinsic motivation by strengthening the feeling of self-determination [5], [8].
- Relevance-oriented teaching: The perceived relevance of content for future medical practice is one of the strongest motivators. Links between basic subjects and clinical applications have been shown to promote autonomous learning [8].
- Structured and transparent learning environment: Clear goals, comprehensible requirements and regular, constructive feedback strengthen the sense of competence. This, in turn, is a key predictor of motivation [5], [6].
- Activating teaching formats: Methods such as problem-oriented learning (POL), team-based learning or simulations promote active participation and a sense of self-efficacy [6].
- Promoting social integration: Group work or mentoring programmes strengthen the sense of belonging and are therefore another cornerstone of motivation [5].

During the clinical phase:

- *Authentic clinical learning opportunities:* Real clinical experiences increase students’ engagement and motivation as they recognise the immediate benefits and relevance [9].
- *Progressive assumption of responsibility:* The concept of “Entrustable Professional Activities” (EPAs) shows that increasing individual responsibility, when appropriately supported, strengthens motivation and professional identity [10].
- *Appreciative and competence-oriented supervision: *Teachers who provide support, formulate clear expectations and give positive, development-oriented feedback increase students’ sense of competence and have a motivating effect [8].
- *Role models and model learning: *Engaged physicians and lecturers serve as motivating role models. Studies show that students are more motivated when they encounter professional, empathetic, and engaging role models [8].
- *Promoting psychological safety: *In learning settings where mistakes can be discussed openly, learning motivation increases and students participate more actively in everyday clinical practice [6].

These motivating factors show that motivation does not arise by chance, but can be actively influenced by the design of the learning environment, the attitude of the teachers, and the educational planning.

This issue features a few articles that address topics that can influence the motivation of students or other stakeholders.

The article by Bauermann et al. deals with entrustment decisions that are related to student autonomy [11]. Melanie Mauch's ideas for interprofessional training can also increase motivation by strengthening the feeling of being part of a team [12]. The same applies to the interprofessional continuing education courses described in the article by Bärbel Wesselborg et al. [13].

## Author’s ORCID

Marjo Wijnen-Meijer: [0000-0001-8401-5047]

## Competing interests

The author declares that she has no competing interests.
